# Assessment of a website aimed at providing information on mental health to secondary school students in Can Tho city, Vietnam

**DOI:** 10.1186/s13034-021-00416-z

**Published:** 2021-11-10

**Authors:** Dat Tan Nguyen, Tam Thi Pham, E. Pamela Wright, Joske Bunders

**Affiliations:** 1 Faculty of Public Health, Can Tho University of Medicine and Pharmacy, Can Tho, Vietnam; 2 Guelph International Health Consulting, Amsterdam, The Netherlands; 3 VU University Athena Institute, Amsterdam, The Netherlands

**Keywords:** Website, Internet, Mental health, Students, Can Tho, Vietnam

## Abstract

**Background:**

The stigmatization of mental health problems is a primary barrier for young people to approach mental health services when they suspect they might have such problems. Nevertheless, the internet has become a common platform on which they are likely to seek information on mental health. As such, this study aimed to explore responses from secondary school students in Can Tho city regarding *suckhoetre.vn* website. This website provided information on health and mental health, and this study assessed the potential relevance, appeal, accessibility, usefulness, and sustainability of the website.

**Methods:**

A cross-sectional study included 643 secondary school students in Can Tho city selected by cluster sampling. Two weeks after the students were introduced to the website, they were invited to evaluate it using an anonymous questionnaire. The Chi-squared test was used to assess the significance of differences in the distribution of selected students’ sociodemographic characteristics.

**Results:**

Most (98.6%) participants visited the website in the two-week period, 74% once or twice a week, the others more often, up to once a day. Their activities included reading information (85.8%), seeking help (17.7%), sharing information (15.5%), giving advice to others (11.0%), and chatting or giving comments (9.8%). Most students rated the website very highly in terms of appeal, relevance, accessibility, and usefulness, and wanted to have access to the website in the future. These findings are positive signals to pursue the possible use of a website on mental health for secondary school students to help raise awareness and support good mental health among adolescents in Can Tho city and beyond.

**Conclusion:**

A website designed to provide information to secondary school students appeared to be a promising way to provide access to information on the topic of mental health. The website should be maintained and introduced widely to students, teachers and parents, with regular evaluation of the effectiveness of this website.

**Supplementary Information:**

The online version contains supplementary material available at 10.1186/s13034-021-00416-z.

## Background

Despite the high risk of mental disorders in adolescence, many young people are not receiving appropriate support and guidance from mental health professionals and the community (including family and school). They possibly lack access to health care and education facilities [1]. In recent years, it has become increasingly common for individuals to seek health information on the internet. Several websites on mental health in different countries, such as *KidsHealth.org*, *youthbeyondblue.com*, and *tamlydoisong.wordpress.com*, appear to play significant roles in improving understanding of mental health and in reducing symptoms of depression [2–4]. Most adolescents in urban areas of high-income countries have access to and make use of online information on mental health, especially those associated with disorders, such as behavioral problems. That young people tend to turn to the internet suggests that they are willing to seek help, and they may be prepared to cooperate with others having similar mental health problems and contribute to forming peer support networks. Internet can improve the accessibility to health care because it has a wide reach [4]. In addition, combining the internet with positive psychology can be considered as an opportunity for health promotion and reduction of mental health symptoms [5].

Vietnam is one of the nations with the most rapidly growing use of the internet, with a greater development than most other countries in the region. It is reported that approximately 50 million Vietnamese, or half of the population, were connected to the internet in 2017 and Vietnam’s penetration rate (54%) was higher than the world average (46.5%) [6]. Two thirds of the internet users accessed it every day, spending on average about 2 h 20 min on weekdays, lesser over weekends. Popular places to access the internet were home (78%), work (31%) and internet shops or cafés (25%) [7].

Previous studies in Vietnam showed that mental health problems of young people were a major concern for health authorities, schools, communities, and families [8–10]. Of particular relevance to this study were the high levels of depression, anxiety, and suicidal ideation among secondary school students in Can Tho city [11]. Although the primary health care system in Vietnam is quite strong, primary mental health care, especially for childhood mental health problems, is non-existent. Despite slight progress, the service environment and response to mental health problems in Vietnam remains largely inadequate. The situation is worse outside the major cities in remote provinces, which lack mental health services and cannot prevent or treat children’s mental health disorders [12]. Stigma related to mental health was highly prevalent in Vietnam, and was reportedly recognized as one of the main factors hindering youth from approaching mental health services [13].

In our previous studies in Vietnam, students, teachers and school health officers had suggested that a web-based information resource on psychology and mental health could be helpful for students [11, 14–16]. In addition, most students reported that they would share their private problems and seek help from a website, if one was available.

For these reasons, students in Vietnam may benefit from anonymous access to information on mental health through a website. Since there was no existing website providing specifically mental health information aimed at that age group, we designed one, taking into account the profile of adolescents living here. There are three main sections in the website—*suckhoetre.vn*, providing students with (1) information on health, (2) information on health-related skills, and (3) health check-up. The main contents were designed based on key topics including general health care, sexual orientation, love and relationships, drug and game addictions, reproductive health, nutrition, and skills to scope with stress in life, as mentioned by students, teachers and parents in previous studies [11, 14–16]. Information in Vietnamese posted on the *suckhoetre.vn* was translated and cited from information on national and international websites, such as kidshealth.org website [17], Mai Huong Daycare Psychiatric Hospital website [18], and Depression website [19]. In addition, current health status screening was integrated in the website, including the General Health Questionnaire [20, 21], the CES-D [22], the Rosenberg self-esteem scale [23, 24], and the Educational Stress Scale for adolescents [25]. Information translated into Vietnamese was checked by the researchers for accuracy and by students of Can Tho University of Medicine and Pharmacy for accessibility to young readers. The design and interface of the website were shaped by the researchers in collaboration with students of Can Tho University of Medicine and Pharmacy. The first draft version was piloted with three school health officers and ten students for feedback, to complete the website. It was launched for public use in 2016, with the aim of meeting the needs of secondary school students for information on mental health and psychological wellbeing in Can Tho city.

After the website was published, we recruited secondary school students to investigate if they appreciated the features of the website, and whether it could be a way forward to recommend education authorities to consider it as a tool to improve students’ mental health. To date, the website remains accessible to public. However, it is not being regularly updated since the completion of the study.

## Methods

### Study design and population

To assess the effectiveness of the website in providing information on mental health, a cross-sectional study was carried out with 643 secondary school students including 318 boys and 325 girls, mean age: 16.99 years. The students were recruited using random sampling in three stages. Firstly, three schools were selected, including the only specialized school in Can Tho city and two of the 23 regular secondary schools, one in an urban and one in a rural district of the city. A specialized secondary school is defined as a secondary school for students with excellent academic results for both developing their talents in certain subjects and ensuring a comprehensive general education [26]. The regular schools had 61 classes of grades 10 to 12 with an average 35 students per class. Two classes from each grade from 10 to 12 were randomly selected by coding all classes in these grades with a number and using Excel software to select two numbers randomly per grade. The specialized secondary school had 26 classes of grades 10 to 12; three classes from these grades were randomly selected. All students in each selected class were invited to participate. The number of valid completed questionnaires was 643, which is 95.4% of the 674 distributed; the other questionnaires were excluded because they were incomplete. Our sample is 643 completed questionnaires submitted by 643 secondary school students. Among these 643 students, 634 students visited the website at surveying time of within 2 weeks after the website’s launch (percentages of students visited and not visited yet are presented in Table 2). The assessment the website was done with 634 students who visited the website. We did not conduct another follow up observation to the 9 students who did not visit the website at that time. Data collection took place in November 2016.

### Data collection

The *suckhoetre.vn* website, its purpose, and the aims of this study were introduced to the students in the selected classes. The students were invited to use the website, then after 2 weeks, they were asked to complete the questionnaire anonymously.

The questionnaire consisted of five components asking the participants about: (1) demographic information on their age, sex, class, school, means of internet connection; (2) their access situation (visited the site or not, visiting activities, number of visits during last seven days); (3) their evaluation of the usefulness, relevance, interest, appeal, and accessibility, using a 5- or 7-point Likert scale ranging from “extremely” to “not at all” with a neutral midpoint, and their willingness to maintain access to the website; and (4) whether they did or would introduce the *suckhoetre.vn* website to others. The questionnaire was composed and completed in Vietnamese. An English version is provided in the Additional file 1.

The measurements on the usefulness, relevance, interest, appeal, and accessibility of the *suckhoetre.vn* website were done for each item from the three main sections of the website: health, skills, and health check-up, to identify which information was most relevant to the students, and which information the students most liked or disliked.

In addition to the data obtained from the questionnaires, we checked the website traffic after it had been up for 1 year, to find out whether it was still being used.

### Statistical analysis

Data were entered into and analyzed by SPSS software 18.0. Data are presented as means ± standard deviation (SD) and analyzed descriptively to determine the demographic characteristics of the study population. The Chi-squared test (χ2) was used to assess the significance of differences in the distribution of selected sociodemographic characteristics. All tests were 2-tailed and a p-value of < 0.05 was considered statistically significant.

## Results

### Socio-demographic characteristics of the sample

Completed questionnaires were submitted by 643 secondary school students of the total 674 distributed, of age ranging from 16 to 20 years with a mean age of 16.99 years. The numbers of boys and girls were almost equal. The number of students from the two regular schools was higher than from the one specialized school. The percentages of students from Grades 10, 11 and 12 were 35.9%, 31.3%, and 32.8%, respectively (Table 1).

**Table 1:** Socio-demographic characteristics of the sample

Characteristics	Total		Boys		Girls		P
	n	%	N	%	n	%	
Total	643	100	318	49.5	325	50.5	
Age (mean ± SD)	16.99 ± 0.86		17.03 ± 0.89		16.96 ± 0.83		0.341^a^
Age group							**0.042**^**b**^
16	228	35.5	114	35.85	114	35.08	
17	203	31.6	87	27.36	116	35.69	
18–20	212	32.9	117	36.79	95	29.23	
Type of school							0.208^b^
Specialized	211	32.8	99	31.1	112	34.5	
Regular	432	67.2	219	68.9	213	65.5	
Grade							**0.027**^**b**^
10	231	35.9	116	36.5	115	35.4	
11	201	31.3	85	26.7	116	35.7	
12	211	32.8	117	36.8	94	28.9	

^a^t-test was used to compare differences in mean of age according to sex

^b^X^2^ was used to compare differences in age groups, types of school and grades according to sex

Almost all of the students (99.2%) had access to electronic devices with internet access on one or more than one type of devices. The mobile phones were the most popular way to access the internet, used by nearly 66% of the student participants. About 50% of them used computer laptops, about 33% used computers and about 20% used iPads.

### Usage of the *suckhoetre.vn* website

After receiving information about *suckhoetre.vn*, nearly all of the students (98.6%) visited the website within the first 2 weeks of its launch. Their main activity on the website was “reading information”, but 10–20% of students also used it for “seeking help”, “sharing information”, “giving advice to others”, and “chatting or giving comments” (Table 2).

**Table 2:** Main activities on *suckhoetre.vn* website within 2 weeks of its launch

Reported Activity	Total % (n)	Sex			School		
		Male % (n)	Female % (n)	p^a^	Specialized % (n)	Regular % (n)	p^a^
Not visited yet	1.4 (9)	1.6 (5)	1.2 (4)	0.487	1.9 (4)	1.2 (5)	0.487
Visited	98.6 (634)	98.4 (313)	98.8 (321)		98.1 (207)	98.8 (427)	
Read information	85.8 (544)	84.0 (263)	87.5 (281)	0.124	88.9 (184)	84.3 (360)	0.075
Search for help	17.7 (112)	20.1 (63)	15.3 (49)	0.067	11.6 (24)	20.6 (88)	0.003*
Share information	15.5 (98)	18.5 (58)	12.5 (40)	0.022*	15.0 (31)	15.7 (67)	0.457
Give advice	11.0 (70)	12.1 (38)	10.0 (32)	0.228	11.1 (23)	11.0 (47)	0.533
Chat/Comment	9.8 (62)	10.5 (33)	9.0 (29)	0.307	12.1 (25)	8.7 (37)	0.113
Other	1.4 (9)	1.3 (4)	1.6 (5)	0.516	2.4 (5)	0.9 (4)	0.133

^a^ X^2^ was used to compare differences in accessing and activities on the website according to sex and types of school

* p < 0.05

Most of the activities were similar among the students. However, the percentage of students from regular shools seeking help was significantly higher than students from the specialized school. Sharing information on the website was more common among boys than among girls. The website was designed to be a medium for students to share information, interact, comment, and/or give advice.

In the week preceding their completion of the questionnaire, more than 75% of the students had visited the website once or twice, while fewer than 10% had visited it more often than that. No significant difference was noted between boys and girls or between specialized and regular schools in terms of the frequency in visiting the website in that week (p > 0.05).

### Students’ evaluation of usefulness, relevance, interest, appeal, accessibility, and possibility to maintain access to the website in the future

When asked to evaluate the website, more than two thirds of the students reported that it was very useful or useful; just 53% said it was “very interesting” or “interesting”; and just 47% referred to it as very or quite appealing. The percentage of students responding that the website was not useful, interesting or attractive was very low, less than 5% in each of the three categories, while higher proportions reported being undecided. A significantly higher percentage of boys reported that the website was very appealing than did girls (Table 3).

**Table 3:** Students’ evaluation of usefulness, interest and attractiveness of the *suckhoetre.vn* website

Item	Total % (n)	Sex			School		
		Male % (n)	Female % (n)	p^a^	Specialized % (n)	Regular % (n)	p^a^
Total sample (n)	634	313	321		207	427	
Usefulness				0.554			0.112
Very much	15.8 (100)	16.9 (53)	14.6 (47)		15.0 (31)	16.2 (69)	
Somewhat	54.6 (346)	54.6 (171)	54.5 (175)		60.4 (125)	51.8 (221)	
Undecided	29.0 (184)	27.5 (86)	30.5 (98)		24.6 (51)	31.1 (133)	
Not really	0.6 (4)	1.0 (3)	0.3 (1)		0 (0.0)	0.9 (4)	
Interest				0.740			0.771
Very much	9.2 (59)	11.8 (37)	6.9 (22)		10.1 (21)	8.9 (38)	
Somewhat	43.8 (278)	44.7 (140)	43.0 (138)		43.5 (90)	44.0 (188)	
Undecided	45.6 (289)	41.9 (131)	49.2 (158)		45.9 (95)	45.4 (194)	
Not really	1.1 (7)	1.6 (5)	0.6 (2)		0.5 (1)	1.4 (6)	
Not at all	0.2 (1)	0.0 (0)	0.3 (1)		0.0 (0)	0.2 (1)	
Appeal				0.024*			0.536
Very much	6.6 (42)	9.3 (29)	4.0 (13)		7.2 (15)	6.3 (27)	
Somewhat	40.4 (256)	41.2 (129)	39.6 (127)		41.1 (85)	40.0 (171)	
Undecided	48.1 (305)	43.5 (136)	52.6 (169)		47.8 (99)	48.2 (206)	
Not really	3.9 (25)	4.8 (15)	3.1 (10)		3.9 (8)	4.0 (17)	
Not at all	0.9 (6)	1.3 (4)	0.6 (2)		0.0 (0)	1.4 (6)	

^a^χ^2^ was used to compare differences in frequency of visiting the website according to sex and types of school

*p < 0.05

Regarding the content on the website, health categories comprised of health care, stress, emotions and feelings, depression, relationships, substance abuse and game addiction, nutrition, and reproductive health. The students reported that the topics of health care, stress, and nutrition were most appropriate to them and they also liked these the most. Other topics were reported as “most approriate” by less than 20% students. The least liked section was the one on drugs and game addiction.

The skills section described tools for learning study skills, soft skills, career skills, handling situations, and protecting skills (how to be safe; how to deal with difficult situations; etc.); these were considered “very suitable” for their own needs by 30.9% of the respondents. The health check-up section (providing ways to measure stress, depression, and self-esteem) was rated by only 18.3% as appropriate for them (Table 4).

**Table 4:** The most appropriate, liked and disliked items on the website

Item	Most appropriate % (n)	Most liked % (n)	Most disliked % (n)
Health			
Health care	57.6 (365)	42.3 (272)	2.8 (18)
Stress	41.6 (264)	25.5 (164)	5.0 (32)
Nutrition	33.3 (211)	22.6 (143)	3.0 (19)
Emotion and feeling	19.6 (124)	11.2 (71)	6.0 (38)
Depression	14.0 (89)	9.0 (57)	6.8 (43)
Reproductive health	12.5 (79)	8.8 (56)	10.9 (69)
Relationship	10.1 (64)	4.3 (27)	9.9 (63)
Stimulant drug and game addiction	9.6 (61)	6.9 (44)	21.1 (134)
Skills	30.9 (196)	21.5 (136)	4.3 (27)
Health check-up	18.3 (116)	8.2 (52)	4.6 (29)

Nearly 80% of the students thought that the website would appeal to their parents and friends. Furthermore, more than 60% felt that the website was very much or somewhat easy to access (Table 5).

**Table 5:** Appeal and accessibility of the *suckhoetre.vn* website

Item	Total % (n)	Sex			School		
		Male % (n)	Female % (n)	P^a^	Specialized % (n)	Regular % (n)	P^a^
Appeal to parents	79.8 (506)	80.2 (251)	79.4 (255)	0.446	81.2 (168)	79.2 (338)	0.316
Appeal to friends	72.9 (462)	73.8 (231)	72.0 (231)	0.333	71.0 (147)	73.8 (315)	0.261
Accessibility				0.920			0.206
Very much	15.5 (98)	15.7 (49)	15.3 (49)		14.0 (29)	16.2 (69)	
Somewhat	49.1 (311)	48.2 (151)	49.8 (160)		54.1 (112)	46.6 (199)	
Undecided	32.0 (203)	31.9 (100)	32.1 (103)		27.5 (57)	34.2 (146)	
Not really	2.7 (17)	3.2 (10)	2.2 (7)		3.9 (8)	2.1 (9)	
Not at all	0.8 (5)	1.0 (3)	0.8 (5)		0.5 (1)	0.9 (4)	

^a^X^2^ was used to compare differences in appeal and accessibility levels of the website according to sex and types of school

When the students were asked whether they would like the website to remain operational, nearly all of them (96.5%) said yes. Most (89%) planned to continue visiting the website in the future; most (88.2%) also agreed they would introduce it to family members and friends, and 88.6% said they would introduce the website to families with members who have mental health problems.

In addition to the data from the questionnaires, we checked the data registered by the website 1 year after its introduction. The website registers the numbers of visits to different items, which revealed that the reproductive health item attracted the highest number of views (850,713), followed by the emotion and feeling section (viewed 442,856 times), and the depression items (viewed 437,252 times). We do not have data on how many people made these visits, nor on who they are, whether they were the target population in the secondary schools or youth in general or indeed parents or others interested in the health of young people. This data does, however, demonstrate that 1 year on, people are still seeking information from the website.

## Discussion

### Use of the mental health information website by secondary school students

Although mental health problems were common among adolescents, barriers including social stigma prevent them from approaching the health services for information and possibly for assistance [27–29]. In the current context of high internet use, we investigated whether information provided by a website designed to attract secondary school students would be a good way to provide them with information about this sensitive topic. After we introduced the website *suckhoetre.vn* to selected secondary schools in Can Tho city, Vietnam, nearly all of the students visited the site at least once or twice a week, and most of them used the website just to read information, which suggests that this is a good approach to give students relatively easy access to information they need. These findings are in line with a report from four focus groups with 20 youths and 20 parents in Danang City, Vietnam in which both parents and students anticipated that the internet would be a useful source for obtaining and sharing information for young people in Vietnam [30]. Most of the youth in our study population had access to the internet at home and very often on smartphones, which would provide the confidentiality they needed. Previous studies on mental health of secondary school students in Can Tho city had recommended for information to be provided through the internet. The present study confirmed that it can indeed be an effective source of information as it has been proven in other countries and proposed in Vietnam earlier [2, 11, 14].

Students in regular schools were significantly more likely to use the website to search for help than students in specialized schools. This might be explained either by different needs among the two groups of students, or by differences in access to information apart from what is available on this website. Further studies need to be carried out to explore this difference.

One of the significant findings was that it was more common for boys than for girls to share information on the website (p < 0.05). This difference might be a result of differences in mental health issues and needs, or of willingness to share such information. Further studies need to be done to capture gender differences pertaining to mental health information seeking to be able to provide in-depth recommendations for designing future website content and program.

### Usefulness, relevance, interest, attractiveness, accessibility, and potential to maintain the *suckhoetre.vn* website

Most of the respondents reported that the website was useful and interesting, confirming that it can be a good source of information for them.

More boys than girls found the website was “very/extremely appealing” (9.3% vs 4.0%), which suggests that gender-specific needs should be considered while designing a website to broaden its appeal. Previous studies have shown that males are more likely to give “extreme” answers in which they strongly agree or disagree [31]. In addition, as mentioned above, boys reported more often that they shared information on the website. This could be a reason for the boys having found the website very appealing.

The topics of health care and psychological and mental health such as stress were considered very appropriate by most of the respondents. In addition, many students agreed that skills including soft skills, career skills, handling situations, and protecting skills were most useful for them. It is important to meet students' needs while providing information related to health care, psychological and mental health and life skills. Therefore, we suggest that in addition to providing access to psychological and mental health information, it may be helpful for schools to include training in the above-mentioned life skills that can help students to cope with daily stress [30].

Students also reported that the health check-up content that helped them learn about depression, and self-esteem on the website was the most suitable and interesting to them. This result suggests that in addition to providing psychological and mental health information on the website, the health check section on the website can be useful to serve as the first source of information on mental health issues that can sensitize them to red flags and need for early intervention.

### Limitations

One limitation of this study was with the sampling process, in which only students aged between 16 and 20 years in senior secondary schools were included. This was, nevertheless an age group with high levels of stress in making the transition to adulthood and a high need for good knowledge about mental health, as shown in previous publications [32, 33]. The study was undertaken in one large city in the south of Vietnam, which may mean that it is not representative for the whole country, although in the current context of media and the internet, the youth in different locations share problems and concerns more often than in the past. One additional limitation of this study was that it only explored male and female differences but did not explore the reactions of students of other genders or sexual orientations. Another limitation is that the data was collected by using a questionnaire specifically designed for the purpose of this study due to unavailability of relevant measures. The young respondents’ personality and identity development are still developing, which could result in fluctuating self-perceptions [34] and thus lead to unreliable reporting. The study did not include a way to corroborate their responses or their actual visits to the website. The data provides information on the students’ use of the website, but did not attempt to find out about students’ actual mental health problems or how the website might help them to seek help, which is, as described above, not readily available everywhere in the country. We are working towards developing and updating material (pictures) for the website. In the meanwhile, images without any copyrights or legal restrictions for their usage have been used.

The majority of students believed that the website has the potential to appeal to parents and friends. In addition, many students said that it was easy for them to access. Many said that they hoped that the website would remain active and that they would return to it in the future. Many students said that they would introduce the website to their family, friends, and families of people with mental health problems. The results suggest that the website has high potential to expand its users to different groups in Vietnam. However, this is one of the first studies in Vietnam on using a website to provide information on psychology and mental health; much research remains to be done to establish the long-term usefulness of such a website.

## Conclusion

The results of this study suggest that a dedicated website can be a useful source of information on health and mental health for secondary school students in urban Vietnam, and may be an effective way to reach students in other parts of the country and the region. The interest shown by the students in the website reinforces previous recommendations that secondary schools in Vietnam should pay more attention to the mental health of their students. More research would be needed to evaluate the long-term usefulness of the website and whether it is appropriate for different school levels in Vietnam.

## Supplementary Information

**Additional file 1.** Assessment of a website aimed at providing information on mental health to secondary school students in can tho city, vietnam.

## Data Availability

The datasets used and analysed during the current study are available from the corresponding author on reasonable request.
