# Supplementary material for: Assessment of a website aimed at providing information on mental health to secondary school students in Can Tho city, Vietnam
Source: Child Adolesc Psychiatry Ment Health. 2021 Nov 10;15:64. doi: 10.1186/s13034-021-00416-z (PMC8582088; doi:10.1186/s13034-021-00416-z)
Supplement: Supplementary file 1 — Additional file 1. Assessment of a website aimed at providing information on mental health to secondary school students in can tho city, vietnam. [file 13034_2021_416_MOESM1_ESM.doc]

**ASSESSMENT OF A WEBSITE AIMED AT PROVIDING INFORMATION ON MENTAL HEALTH TO SECONDARY SCHOOL STUDENTS IN CAN THO CITY, VIETNAM**

**Dat Tan Nguyen^1*^, Tam Thi Pham^1^, E. Pamela Wright^2^, Joske Bunders^3^**

*^1^ Faculty of Public Health, Can Tho University of Medicine and Pharmacy, Vietnam*

*^2^Guelph International Health Consulting, Amsterdam, the Netherlands*

*^3^ VU University Athena Institute, the Netherlands*

*Corresponding author:

Dat Tan Nguyen, Faculty of Public Health, Can Tho University of Medicine and Pharmacy, Can Tho city, Vietnam

E-mail: [ntdat@ctump.edu.vn](mailto:ntdat@ctump.edu.vn)

**THE QUESTIONNAIRE FOR ASSESSMENT OF THE PARTICIPATION USE WEBSITE SUCKHOETRE**

**WEBSITE** [**http://www.suckhoetre.vn/**](http://www.suckhoetre.vn/)

The dear students,

Please provide information on request or answer by circling the appropriate number corresponding with you for the questions below!

| **ID** | **Questions** | **Answers** | **Notes** |
| --- | --- | --- | --- |
|  | What year were you born? | ….................. |  |
|  | Are you male or female? | 1. Male  2. Female |  |
|  | Which grade are you in? | ……………… |  |
|  | Have you received information introducing about the website suckhoetre.vn before? | 1. Yes 2. No |  |
|  | You have ever visited suckhoetre.vn website yet? | 1. Yes  2. No,  Reasons? ………………....................  …………………………………….. | If "no" move to question 10 |
|  | Once on the website suckhoetre.vn, what activities do you have? (you can circle more reviews) | 1. Look for information  2. Find helpers  3. Share information  4. Provide advice to help others  4. Chat/comment  5. Other activities (specify) …………… |  |
|  | In the past week, how did your times to visit the website suckhoetre.vn like? | 1. Everyday  2. From 3 times and over  3. 1-2 times  4. None |  |
|  | Could you please to evaluate the usefulness of this suckhoetre.vn site? | 1. Very useful  2. Useful  3. No idea  4. Not helpful  5. Very not helpful |  |
|  | Could you please to evaluate the favorite level of this suckhoetre.vn site? | 1. Like so much  2. Like  3. No idea  4. Don’t like  5. Very dislike |  |
|  | Could you please to evaluate the attractive level of this suckhoetre.vn site? | 1. Very attractive  2. Attractive  3. No idea  4. Not attractive  5. Very not attractive |  |
|  | According to you, can this website appeal to your parents to access it? | 1. Yes 2. No   Reason why? ………………....................  …………………………………….. |  |
|  | According to you, can this website appeal to your friends to access it? | 1. Yes 2. No   Reason why? ………………....................  …………………………………….. |  |
|  | Could you please to evaluate the easiness of this suckhoetre.vn site? | 1. Very easy  2. Easy  3. No idea  4. Not easy  5. Very not easy |  |
|  | According to you, what information on the website is suitable to you? (can choose more than one answer) | 1. Health  Reason why? ………………....................  2. Stress  Reason why? ………………....................  3. Depression  Reason why? ………………....................  4. Substance/game addiction  Reason why? ………………....................  5. Reproductive health  Reason why? ………………....................  6. Nutrition  Reason why? ………………....................  7. Health check  Reason why? ………………....................  8. Skills  Reason why? ………………....................  9. News  Reason why? ……………….................... |  |
|  | According to you, what is your most favorite contents or topics on the suckhoetre.vn website? (can choose more than one answer) | 1. Health  Reason why? ………………....................  2. Stress  Reason why? ………………....................  3. Depression  Reason why? ………………....................  4. Substance/game addiction  Reason why? ………………....................  5. Reproductive health  Reason why? ………………....................  6. Nutrition  Reason why? ………………....................  7. Health check  Reason why? ………………....................  8. Skills  Reason why? ………………....................  9. News  Reason why? ………………....................  10. Other (specify): ………………………  ……………………………………………. |  |
|  | According to you, what is your most dislike contents or topics on the suckhoetre.vn website? (can choose more than one answer) | 1. Health  Reason why? ………………....................  2. Stress  Reason why? ………………....................  3. Depression  Reason why? ………………....................  4. Substance/game addiction  Reason why? ………………....................  5. Reproductive health  Reason why? ………………....................  6. Nutrition  Reason why? ………………....................  7. Health check  Reason why? ………………....................  8. Skills  Reason why? ………………....................  9. News  Reason why? ………………....................  10. Other (specify): ………………………  ……………………………………………. |  |
|  | Will you continue to visit the website suckhoetre.vn in the future? | 1. Yes  2. No  Reason why? ………………....................  …………………………………….. |  |
|  | Do you want the web suckhoetre.vn to continue to maintain operation in the future? | 1. Yes  2. No  Reason why? ………………....................  …………………………………….. |  |
|  | Does your family have a computer or another device connected with internet? | 1. Yes  2. No |  |
|  | If yes, what are your internet connection means? (can choose more than one answer) | 1. Desktop  2. Laptop  3. Mobile phone  4. Ipad or tablets  5. Others (specify): ……………………… |  |
|  | Are you willing to introduce the suckhoetre.vn website to your family members? | 1. Yes  2. No  Reason why? ………………....................  …………………………………….. |  |
|  | Are you willing to introduce the suckhoetre.vn website to your family members? | 1. Yes  2. No  Reason why? ………………....................  …………………………………….. |  |
|  | If your relatives or friends having mental health like stress, depression… are you willing to introduce the suckhoe.vn to them? | 1. Yes,  Reason why? ………………………  ………………………………………  2. No,  Reason why? …….....................  ……………………………………… |  |
|  | Do you know other websites providing information about mental health? | 1. Yes  Please specify: …………………………….  ……………………………………………..  2. No |  |
|  | If yes, how could you please compare the website suckhoetre.vn with other websites? | 1. This Website is much better  2. This Website is better  3. Same to other websites  4. This Website is worse |  |
|  | Do you like to find information through a website? | 1. Yes  2. No  Reason why: ……………………………..  …………………………………………… |  |
|  | Do you like to find this information via the media (source) of the following? | 1. Books 2. Friends 3. Teachers 4. Parents 5. Health staffs 6. Other (specify): ………………….. |  |

Would you please spend little time to give us your feedback to this website more enjoyable?

1. According to you, how does the current appearance of this website look like? There is a need to improve more? And how to improve?

………………………………………………………………………………………………

………………………………………………………………………………………………

………………………………………………………………………………………………

………………………………………………………………………………………………

………………………………………………………………………………………………

………………………………………………………………………………………………

………………………………………………………………………………………………

2. According to you, how does the current contents of this website look like? There is a need to improve more? And how to improve?

………………………………………………………………………………………………

………………………………………………………………………………………………

………………………………………………………………………………………………

………………………………………………………………………………………………
